# My heart will go on – quantitative content analysis of donor and donation images in patients after heart transplantation

**DOI:** 10.3389/fpsyg.2026.1813966

**Published:** 2026-09-08

**Authors:** Leoni Wahlers, Nora M. Laskowski, Georg Halbeisen, Katharina Tigges-Limmer, Georgios Paslakis

**Affiliations:** 1 Department of Psychosomatic Medicine and Psychotherapy, LWL-University Hospital, Ruhr-University Bochum, Bochum, Germany; 2 Clinic for Thoracic and Cardiovascular Surgery, Herz- und Diabeteszentrum NRW, Ruhr-University Bochum, Bad Oeynhausen, Germany

**Keywords:** DDI, donor and donation images, donor fantasies, heart failure, heart insufficiency, heart transplantation, organ fantasies

## Abstract

**Introduction:**

Heart transplantation (HTX), a treatment for end-stage heart failure, is accompanied by substantial psychological and physical strain for recipients. Many individuals develop Donor and Donation Images (DDI), referring to the thoughts and emotions directed toward the donor and/or the transplanted organ. The present study aims to systematically quantify the content dimensions of DDI and to explore their associations with psychological well-being and perceived organ acceptance.

**Methods:**

A total of 407 adult post-HTX patients participated in the survey. A self-developed questionnaire assessed 12 content categories of DDI, together with their perceived consequences. Descriptive analysis, gender comparisons, and exploratory factor analysis (EFA) were conducted to examine the underlying structure of DDI. Linear regressions and Spearman correlations were used in an exploratory manner to investigate statistical associations between DDI factors and psychological and emotional outcomes.

**Results:**

The analysis of 402 complete datasets yielded three content factors: Social, Sex/Age, and Identity, capturing different facets of DDI. Women were more likely than men to contemplate the donor’s gender, age, and personality traits. Regression analysis showed small but statistically significant associations between the Social factor and perceived changes in personality and behavior, as well as between the Social and Sex/Age factors and positive emotional consequences. Across all models, the explained variance was low. No meaningful associations were observed with organ acceptance.

**Discussion:**

This study represents the first quantitative attempt to structure the contents of DDI and to examine their psychological correlates in a large cohort of HTX recipients. While the observed associations were modest, the findings contribute to a more nuanced understanding of how recipients engage cognitively and emotionally with their donor. The results underscore the need for further research, including instrument refinement and longitudinal designs, to better capture the complexity of DDI and their potential relevance for psychosocial care in transplantation.

## 1 Introduction

Heart transplantation (HTX) is the treatment of choice for terminal heart failure and severe, irreversible cardiac injury. In 2024, more than 4,500 hearts were transplanted in the United States (Organ Procurement and Transplantation Network, 2025), and 350 in Germany (Deutsche Stiftung Organtransplantation, 2025). The procedure is known to be accompanied by substantial psychological and physical strain for both recipients and their relatives (Tigges-Limmer et al., 2005, 2017), with many patients experiencing a persistent sense of life threat throughout the transplantation process (Tigges-Limmer and Gummert, 2010; Ladwig et al., 2014).

Emotional experiences surrounding HTX can affect postoperative well-being, may have a profound effect on the recovery process, and are associated with a reduced quality of life (Goetzmann et al., 2009). One potential source of psychological burden is the recipients’ thoughts and feelings about the donor (Goetzmann et al., 2009). This phenomenon, recently termed Donor and Donation Images (DDI) (Laskowski et al., 2023, 2025), encompasses all rational and non-rational cognitions and emotions directed toward the donor and/or the transplanted organ. Patients may, for example, wonder who the donor was, how they died, or what personality traits they possessed (Neukom et al., 2012).

In Germany, where the present study was conducted, donor–recipient confidentiality is legally protected under the German Transplantation Act (Transplantationsgesetz; TPG). Personal data identifying donors and recipients must not be disclosed (§14 TPG), and although anonymized correspondence between recipients and donor families is permitted, the identities of all parties must remain protected (§12a TPG).

Existing knowledge about DDI is largely based on qualitative research. Ivarsson et al. (2013) have shown that heart and lung recipients frequently reflect on characteristics such as the donor’s gender and age, and often experience profound gratitude for the opportunity to regain a near-normal life. Case series among lung transplant patients have described assumptions about the donor’s family situation, circumstances of death, with some patients even reporting very specific assumptions on these topics (Neukom et al., 2012). Interestingly, some patients after HTX and kidney transplantation reported subjectively experiencing personality changes after receiving a new organ (Bunzel et al., 1992; Sanner, 2003), or that a new person lived within their body influencing their behavior and lifestyle (Goetzmann, 2004). Some patients even reported some sort of relationship with the donor and communicating with them (Goetzmann, 2004). Eichenlaub et al. (2021) found that around 13% of patients following lung transplantation believed that the donor became part of their “inner world.” Older reports describe concerns about receiving organs from donors with particular characteristics, such as sexual orientation or age (Basch, 1973).

Taken together, these qualitative observations suggest that HTX recipients may form vivid and personally meaningful representations of their donors, sometimes accompanied by a perceived sense of closeness or influence. However, these impressions have not yet been systematically quantified, and it remains unclear whether DDI contribute to psychological distress, serve as a coping mechanism, or relate to other aspects of postoperative adjustment. Given the associations between psychological distress and poor organ integration reported in previous research (Hennemann et al., 2021), understanding the content and emotional relevance of DDI may help elucidate key recovery processes following HTX.

In a recent study, we provided the first systematic investigation of the occurrence of DDI in HTX patients (Laskowski et al., 2025), showing that DDI are highly prevalent (91%), particularly shortly after and shortly before transplantation. DDI were associated with psychological strain, though they may also reflect heightened emotional activation. Whether DDI operate as stressors, coping responses, or both remains an open question.

Overall, empirical knowledge on DDI is limited, and further research is required, particularly regarding the specific contents of DDI and their potential psychological correlates. Since earlier qualitative studies have noted gender-specific aspects, especially regarding men’s concerns about receiving organs from female donors (Bunzel et al., 1992; Sanner, 2003), we additionally explored gender differences. As an extension of our previous work, the present study aimed to identify the underlying content dimensions of DDI using a quantitative approach for the first time. We also examined how these content dimensions may relate to perceived personality and behavioral changes, emotional responses, and organ acceptance. Given the absence of comparable quantitative investigations, all analyses were conducted in an exploratory manner.

## 2 Materials and methods

### 2.1 Sample

Participants were adult patients who had undergone HTX (*N* = 407, see also Laskowski et al., 2025). The original study included all adult patients with sufficient German language proficiency from the existing pool of individuals who had undergone HTX at the Herz- und Diabeteszentrum NRW and were registered as alive (*N* = 1,023). The survey was distributed in January 2023. A total of 416 individuals completed the questionnaire (response rate: 40.7%); after exclusion of one underage participant and eight participants who completed less than 50% of the DDI-relevant questions, the final sample comprised *N* = 407 participants. Further details on recruitment and study procedures are provided in Laskowski et al. (2025). All participants provided written informed consent prior to their participation in the study. The study adhered to all ethics statements and was reviewed and approved by the Ethics Committee of the Medical Faculty of the Ruhr-University Bochum (AZ 2022–959). The study was prospectively registered on AsPredicted (#112370).

### 2.2 Questionnaire

A self-developed questionnaire, previously described in detail in Laskowski et al. (2025), was used to assess DDI. Participants self-reported their gender using the categories male, female, diverse, queer, or prefer not to disclose. As no validated instrument for measuring DDI currently exists, the development of an initial exploratory questionnaire was necessary to enable a systematic quantitative assessment of this phenomenon. Based on recurring themes from qualitative studies (e.g., Goetzmann, 2004; Kaba et al., 2005; Ivarsson et al., 2013), we assessed DDI contents across 12 categories: donor’s sex, age, sexual orientation, origin, religion, personality, life history, lifestyle, profession, education, family situation, and cause of death. For each category, patients indicated whether they had ever contemplated the respective aspect (yes/no) and could optionally further describe their thoughts in an open-ended text field (“Yes, specifically […]”).

Open-ended responses were used solely to further describe participants’ specifications following a “yes” response to a DDI content category. Responses with the same or equivalent content were grouped into descriptive categories and frequencies were calculated. All responses could be unambiguously assigned to these categories. No formal qualitative content analysis was conducted.

To examine perceived consequences of DDI, participants responded to items assessing personality- and behavior-related perceptions (e.g., “Sometimes I think that my personality has changed because of the transplanted heart”) as well as changes described by others (e.g., “People around me tell me that I have changed as a result of the heart transplant”). Additional items assessed perceived consequences for organ acceptance (e.g., “Thinking about my donor helps me to accept the transplanted heart as my own”) and emotional responses. Emotional consequences included neutral, negative (e.g., “The feelings towards the donor depress me”), and positive items (e.g., “I like to think about the donor”).

### 2.3 Statistical analysis

Analysis was conducted using Python (Python Software Foundation, 2024) version 3.12.4 (packages: pandas version 2.2.2, The pandas development team, 2020; seaborn version 0.13.2, Waskom, 2021; matplotlib version 3.9.0, Hunter, 2007) and SPSS version 28.0.1.1 (IBM Corp, 2022). Data are available from the corresponding author, NML, upon reasonable request.

Descriptive statistics (absolute and percentage frequencies) were calculated for all DDI content categories. Gender differences were examined using chi-square tests (χ^2^) with Cramer’s V as the effect size (0.10 = small, 0.30 = medium, > 0.50 = large) (Cohen, 1988). Due to low cell counts in some categories, gender comparisons refer only to the presence of thoughts in the broader categories, not to the specific qualitative elaborations.

Given the limited existing knowledge about DDI contents, an exploratory analytic approach was adopted. An Exploratory Factor Analysis (EFA) was conducted on the 12 dichotomous content categories using a tetrachoric correlation matrix. Varimax rotation was applied to enhance interpretability (Ferguson and Cox, 1988). Sampling adequacy was assessed via the Kaiser–Meyer–Olkin (KMO) measure (≥ 0.50 acceptable); values approaching 1.0 indicate high adequacy (Dziuban and Shirkey, 1974) and the Measure of Sampling Adequacy (MSA) for each variable (minimum 0.60). The number of factors was determined using parallel analysis.

All variables were retained regardless of loading magnitude, based on theoretical considerations (even moderately loading items may represent relevant DDI content domains) and empirical reasoning (low-loading items may capture subtler but meaningful content variations). Model reliability was evaluated using the Tucker–Lewis Index.

Items assessing consequences of DDI were grouped by thematic domain, and mean indices were computed. Indices included changes in personality and behavior (6 items), consequences for organ integration (6 items), positive emotional responses (5 items), and negative emotional responses (3 items).

To explore statistical associations, linear regression models were calculated with the factor scores from the EFA entered as predictors and the respective DDI consequence indices as dependent variables. Given the exploratory nature of the study, these analyses are interpreted as preliminary associations rather than predictive models. Spearman correlations were calculated among the EFA-derived factors and between the factors and consequence indices.

## 3 Results

### 3.1 Sample

A total of 407 patients participated in the study. Data sets with more than 20% missing responses in the DDI content items were excluded (*n* = 5), resulting in 402 datasets for the main analysis. For the EFA, all rows with missing values in the relevant variables were removed, yielding a subsample of *N* = 385. A description of the sample is provided in Table 1 (see also Laskowski et al., 2025 for additional details).

**Table 1:** Sample description.

Variable	*N* (%)
Gender	
Male	294 (72.4)
Female	111 (27.3)
Diverse	1 (0.2)
Mean age (SD, Range)	57.91 (14.01, 18–90)
Mean age at transplantation (SD, Range)	48.17 (14.24, 1–71)
School years	
< 12 years	224 (56.3)
≥ 12 years	174 (43.7)
Partnership status	
With partner	304 (77.9)
Without partner	86 (22.1)

Table 1 refers to the enrolled sample (*N* = 407). Missing values: gender, *n* = 1; school years, *n* = 9; partnership status, *n* = 17. No values were missing for age or age at transplantation.

### 3.2 Categorical analysis of the contents of DDI

**Figure 1:**
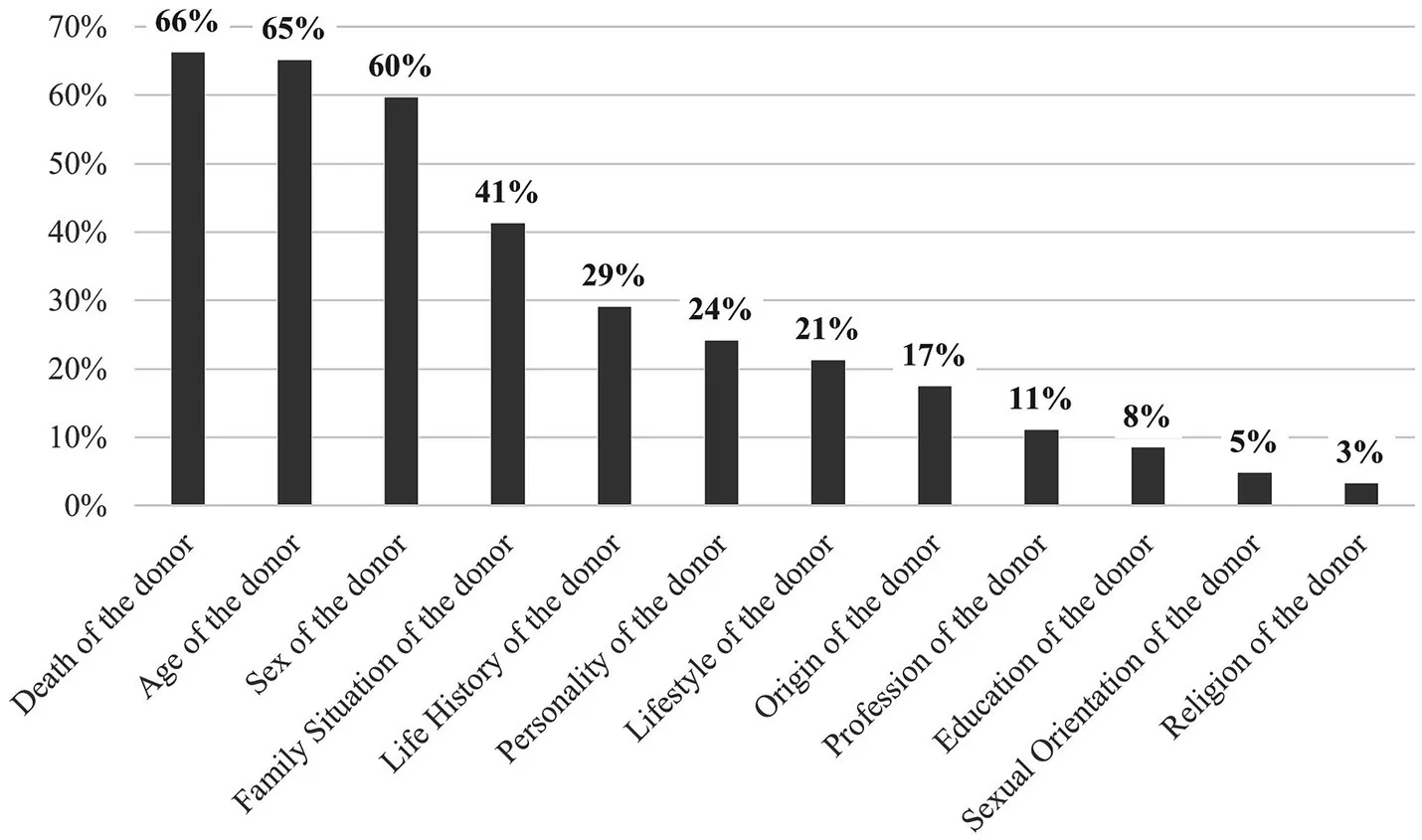
Frequency of different aspects of donor and donation images.

Figure 1 illustrates the frequency of DDI content categories. More than half of the respondents reported having thoughts about the donor’s gender (60%), age (65%), or cause of death (66%). The donor’s family situation was also frequently considered (41%), whereas aspects such as sexual orientation (5%) and religion (3%) were mentioned comparatively rarely.

A total of 241 respondents reported having thought about the donor’s sex (59.8%; no thoughts *n* = 162, 40.2%; 4 missing responses). Among those who had contemplated this aspect, 59.4% assumed a same-sex donor and 40.6% an opposite-sex donor (*n* = 76 vs. *n* = 52; 274 participants provided no assumption). Women were significantly more likely than men to report thinking about the donor’s sex (*N* = 400; χ2[1] = 9.18, *p* = 0.002, V = 0.15). However, when examining assumptions about the donor’s sex (same-sex vs. opposite-sex) in relation to the recipient’s gender (dichotomized male/female), no significant gender difference emerged (χ2[1] = 2.12, *p* = 0.146, V = 0.15).

Table 2 provides an overview of respondent’s assumptions regarding the donor’s age. A total of 261 individuals (65.3%; no thoughts: *n* = 139, 34.8%, 7 missing responses) indicated that they had considered the donor’s age. The majority presumed that the donor was younger than themselves. Women were more likely than men to think about the donor’s age (*N* = 397; χ2[1] = 6.61, *p* = 0.010, V = 0.13).

**Table 2:** Presumptions about the donor’s age in relation to the HTX recipient’s age.

Presumed age of the donor (253 missing responses)	*n* (%)
Same age	34 (22.8)
Younger	90 (60.4)
Younger or older	10 (6.7)
Younger or same age	7 (4.7)
Older	8 (5.4)

The response categories were derived from the open-ended free-text answers provided by participants who indicated having thought about the donor’s age.

Twenty participants reported having thought about the donor’s sexual orientation (5.0%; no thoughts: *n* = 380, 95.0%, 7 missing responses). Of these, 11 further specified their assumptions, all indicating that they presumed their donor to be heterosexual. No significant gender differences emerged in the likelihood of thinking about the donor’s sexual orientation (*N* = 397; χ2[1] = 2.87, *p* = 0.090, V = 0.09).

With regard to religion, 15 respondents (3.7%; no thoughts: *n* = 389, 96.3%; 3 missing responses) indicated having reflected on this aspect. Only three participants specified their assumptions: two presumed a Christian donor, and one assumed the donor shared their own religion. Due to the very small number of specific responses, no gender comparison was conducted.

Seventy respondents (17.4%; no thoughts: *n* = 332, 82.6%; 5 missing answers) reported having considered the donor’s origin. The term “origin” was not further specified in the questionnaire and was therefore open to participants’ interpretation. Among these, 51.6% assumed that the donor came from outside Germany (*n* = 16), 25.8% presumed the donor was German (*n* = 8), and 22.6% were unsure (*n* = 7). No significant gender differences were observed (*N* = 400; χ2[1] = 2.87, *p* = 0.090, V = 0.09).

A total of 79 respondents (24.1%; no thoughts: n = 305, 75.9%; 5 missing responses) reported having thought about the donor’s personality. Women were more likely than men to contemplate this aspect (*N* = 400; χ2[1] = 5.94, *p* = 0.015, V = 0.12). Table 3 summarizes the assumptions regarding personality characteristics.

**Table 3:** Perception of the donor’s personality.

Presumed personality characteristics of the donor (364 missing responses)	*n* (%)
Negative	2 (5.3)
Positive	26 (68.4)
Similar to own personality	4 (10.5)
Other assumptions^*^	6 (15.8)

The response categories were derived from the open-ended free-text answers provided by participants who indicated having thought about the donor’s personality. ^*^Other assumptions: interest in cars, introvert or extrovert, left-handed, diverse interests, unfulfilled dreams.

Thoughts about the donor’s life history were reported by 117 respondents (29.3%; no thoughts: *n* = 283, 70.8%; 7 missing responses). These assumptions mainly referred to aspects related to relatives or friends (37.9%, *n* = 11; 373 missing responses), health (24.1%, *n* = 7), interests or hobbies (13.8%, *n* = 4), the circumstances of the donor’s death (17.2%, *n* = 5), and the donor’s work situation (6.9%, *n* = 2). No gender differences were observed (*N* = 397; χ2[1] = 2.86, *p* = 0.091, V = 0.09).

Similar themes emerged for presumed lifestyle, which was considered by 85 individuals (21.2%; no thoughts: *n* = 316, 78.8%; 6 missing responses). Among these respondents, 53.1% reported thoughts related to health or fitness (*n* = 17; 370 missing responses), 15.6% mentioned assumptions about addictive behaviors or vices (*n* = 5), and 31.3% reported other types of thoughts (*n* = 10; including: balanced lifestyle, good lifestyle, individual lifestyle, “normal” lifestyle, fun-loving, respectable lifestyle, unpredictable, soccer player, wild lifestyle). No gender differences were found (*N* = 398; χ2[1] = 0.39, *p* = 0.532, V = 0.03).

Forty-four respondents (11.1%; no thoughts: *n* = 354, 88.9%; 9 missing responses) reported having contemplated the donor’s profession. No gender differences were detected (*N* = 396; χ2[1] = 0.46, *p* = 0.499, V = 0.03). The specific assumptions mentioned were (each *n* = 1): similar profession, craftsman, profession in the social sector, cook, truck driver, cleaner, singer, athlete, and student.

Thoughts about the donor’s level of education were reported by 38 individuals (9.4%; no thoughts: *n* = 368, 90.6%; 1 missing response). Four participants assumed that the donor had a degree, and another four presumed an intermediate level of education (392 missing responses). No gender differenced were observed (*N* = 400; χ2[1] = 1.13, *p* = 0.287, V = 0.04).

More respondents (*n* = 165, 41.3%; no thoughts: *n* = 235, 58.8%; 7 missing responses) reported having contemplated the donor’s family circumstances. Table 4 provides an overview of the presumed family status. No significant gender differences were found (*N* = 398; χ2[1] = 3.41, *p* = 0.065, V = 0.09). Most respondents assumed that the donor had a family of their own (84.4%, *n* = 54).

**Table 4:** Perception of the donor’s family status.

Presumed family status of the donor (336 missing responses)	*n* (%)
Similar to own family status	1 (1.6)
With family	54 (84.4)
With partner	5 (7.8)
Single	4 (6.3)

The response categories were derived from the open-ended free-text answers provided by participants who indicated having thought about the donor’s family status.

More than half of the respondents (*n* = 257, 63.8%; no thoughts: *n* = 146, 36.2%; 4 missing responses) reported having thoughts about the donor’s cause of death. Among those who specified their assumptions, most presumed that the donor had died in an accident (78.6%, *n* = 92; 285 missing responses). Some respondents were unsure whether the cause was an accident or an illness (15.4%, *n* = 18), while 4.3% assumed the donor died due to illness (*n* = 5). Two individuals (1.7%) believed that the donor had died by suicide. No gender differences were found (*N* = 398; χ2[1] = 1.83, *p* = 0.176, V = 0.07).

### 3.3 Exploratory factor analysis of DDI content

The tetrachoric correlations indicated significant associations among all 12 content variables (*p* < 0.001; see Supplementary Table S1). All variables showed acceptable MSA values above 0.60 (range: 0.66–0.91; Supplementary Table S2) and were therefore retained for the exploratory analysis. The KMO value of 0.79 suggested good overall sampling adequacy.

**Figure 2:**
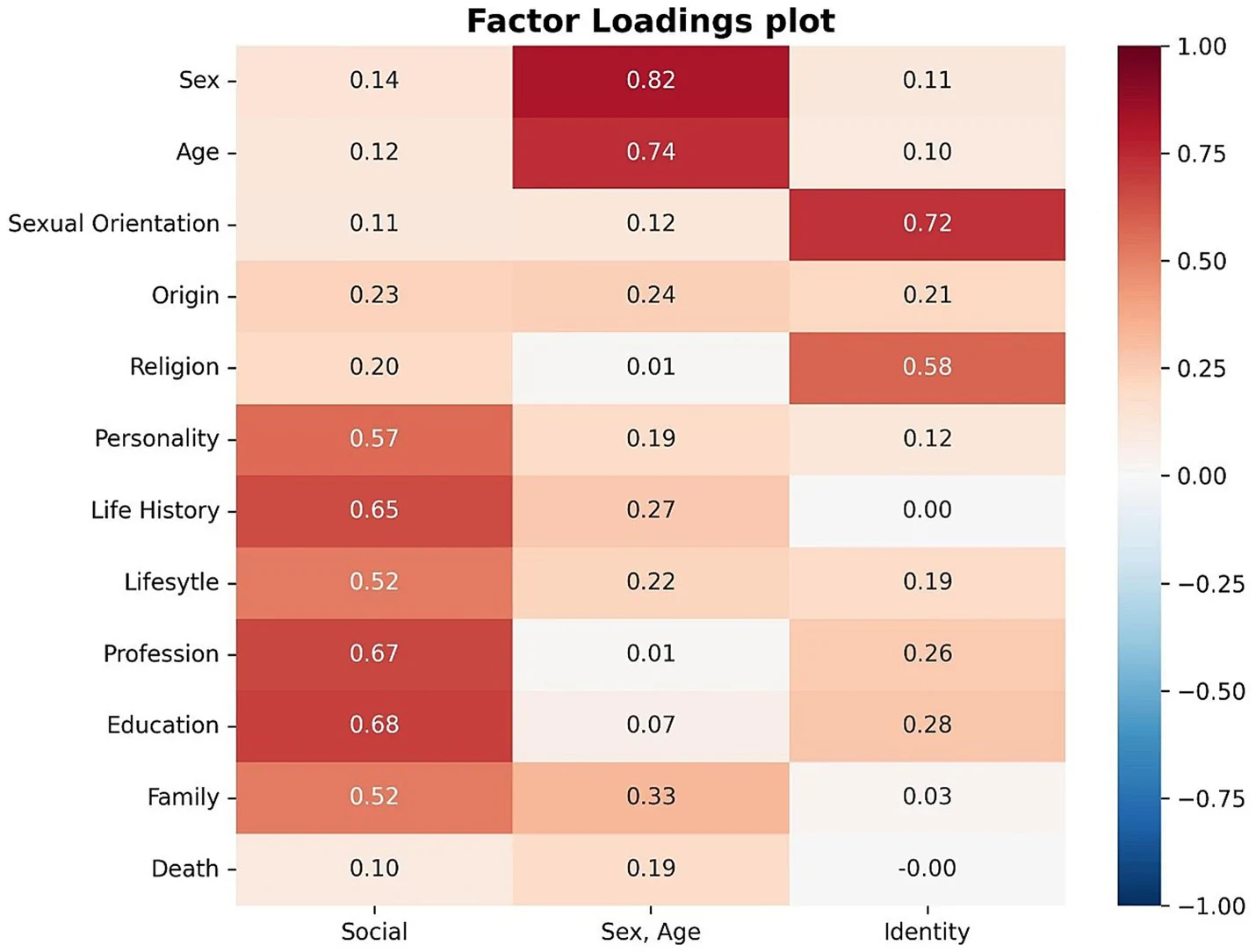
Heat map of factor loadings and item assignments.

An EFA was conducted to provide an initial structural mapping of DDI content (rather than to establish a psychometrically optimized measurement model). Parallel analysis indicated a three-factor solution (Supplementary Figure 1). The corresponding eigenvalues 3.19 (factor 1), 0.70 (factor 2), and 0.43 (factor 3). Figure 2 displays the factor loadings and item assignments. Items related to Personality, Life History, Lifestyle, Profession, Education, and Family Situation loaded on the first factor, which we labeled Social based on item content. Items related to the donor’s Sex and Age loaded on the second factor (Sex, Age). Items assessing Sexual Orientation and Religion loaded on a third factor (Identity). The labeling of the factors was based on the evaluation of the items’ content.

The items Origin and Cause of Death showed loadings below 0.50 and therefore did not clearly load onto any factor. The weak loadings of Origin and Cause of Death suggest that these items may represent qualitatively distinct considerations that are not easily integrated into broader donor-related schemas. Rather than reflecting measurement noise alone, these aspects may capture situational or reality-based cognitions that differ from more relational or symbolic donor representations. Their retention was therefore theoretically motivated, but they were not emphasized in subsequent factor-based interpretations.

The proportion of explained variance was 47% for the Social factor, 31% for the Sex, Age factor, and 22% for the Identity factor. The Tucker-Lewis index was 0.86, indicating a high model fit for an exploratory analysis. Cronbach’s alpha coefficients were 0.79 for the Social factor and 0.79 for the Sex, Age factor. The Identity factor showed limited internal consistency (α = 0.62) and should therefore be interpreted with particular caution; it is best understood as an initial, tentative clustering of content domains rather than a stable latent construct. Overall internal consistency was 0.79. Spearman correlations among the factors are presented in Supplementary Table S3.

### 3.4 Perceived sympathy and similarity to the donor

Only part of the sample responded to the item assessing subjective liking (*N* = 268) and perceived similarity to the donor (*N* = 224). Among those who answered the question on sympathy, most respondents perceived their donor as either very likeable (47.8%, *n* = 128) or rather likeable (51.9%, *n* = 139). Only one person (0.4%) reported disliking their donor.

A similar pattern emerged for perceived similarity. In total, 19.2% (*n* = 43) stated that they considered the donor to be very similar to themselves, and 56.3% (*n* = 126) reported perceiving the donor as rather similar. Fewer respondents indicated that they did not think the donor was similar (17.9%, n = 40), and 6.8% (*n* = 15) reported no perceived similarity at all.

### 3.5 Perceived consequences of donor and donation images

#### 3.5.1 Perceived changes in personality and behavior

More than half of the respondents indicated that neither they themselves nor people around them perceived any personality changes related to the transplanted heart (see Table 5). Approximately 10% of respondents, however, reported experiencing personality changes that were also noticed by others. A similar pattern was observed for perceived changes in behavior, although the distribution of responses was somewhat more variable.

**Table 5:** Perceived changes in personality and behavior.

Statement (missing responses)	Answer scale			
	Strongly agree *n* (%)	Agree *n* (%)	Disagree *n* (%)	Strongly disagree *n* (%)
People around me tell me that I have changed as a result of the heart transplant (23)	42 (10.9)	51 (13.3)	59 (15.4)	232 (60.4)
Sometimes I think that my personality has changed because of the transplanted heart (14)	38 (9.7)	58 (14.8)	62 (15.8)	235 (59.8)
The transplanted heart had no influence on my personality (18)	180 (46.3)	56 (14.4)	52 (13.4)	101 (26.0)
My behavior toward other people has changed since I received the donor organ (19)	72 (18.6)	99 (25.5)	62 (16.0)	155 (39.9)
Sometimes I think that my behavior has changed because of the transplanted heart (20)	74 (19.1)	88 (22.7)	62 (16.0)	163 (42.1)
The transplanted heart had no influence on my behavior (20)	132 (34.1)	55 (14.2)	70 (18.1)	130 (33.6)

#### 3.5.2 Perceived influence of donor and donation images on organ acceptance

Participants generally reported little difficulty accepting the transplanted heart (Table 6). Most participants disagreed with statements suggesting the heart felt foreign or difficult to accept. Overall, participants did not perceive thinking about the donor as having a negative influence on organ acceptance; if anything, thoughts about the donor were more often described as helpful rather than complicating.

**Table 6:** Perceived consequences of donor and donation images on organ acceptance.

Statement (missing responses)	Answer scale			
	Strongly agree *n* (%)	Agree *n* (%)	Disagree *n* (%)	Strongly disagree *n* (%)
I have difficulty accepting the transplanted heart as my own (13)	8 (2.0)	9 (2.3)	52 (13.2)	325 (82.5)
The transplanted heart feels “foreign” (9)	3 (0.8)	9 (2.3)	35 (8.8)	351 (88.2)
I would never describe my heart as “foreign” (11)	227 (57.3)	62 (15.7)	30 (7.6)	77 (19.4)
Sometimes I have the feeling that all the thinking about the donor is not good for the transplanted heart (25)	19 (5.0)	31 (8.1)	54 (14.1)	278 (72.8)
Thinking of my donor helps me to accept the transplanted heart as my own (24)	98 (25.6)	84 (21.9)	54 (14.1)	147 (38.4)
Thinking about my donor makes it difficult for me to accept the transplanted heart as my own (24)	12 (3.1)	15 (3.9)	31 (8.1)	325 (84.9)

#### 3.5.3 Perceived emotional consequences of donor and donation images

Most participants indicated that DDI did not make them feel depressed and did not lead to rumination (Table 7). However, approximately half of the respondents reported that they did not particularly like thinking about the donor. A similar pattern emerged for items assessing whether DDI had a supportive or coping-related effect, with responses distributed across the answer categories.

**Table 7:** Perceived emotional consequences of donor and donation images.

Statement (missing responses)	Answer scale			
	Strongly agree *n* (%)	Agree *n* (%)	Disagree *n* (%)	Strongly disagree *n* (%)
Neutral emotions				
My feelings toward the donor have changed over time (41)	22 (6.0)	30 (8.2)	54 (14.8)	260 (71.0)
When I think of the donor, I do not feel any particular emotion (41)	39 (10.7)	74 (20.2)	88 (24.0)	165 (45.1)
When I think of the donor, I tend to feel indifference (38)	9 (2.4)	25 (6.8)	65 (17.6)	270 (73.2)
Positive emotions				
I like to think about the donor (36)	68 (18.3)	121 (32.6)	76 (20.5)	106 (28.6)
Thinking about the donor gives me strength in challenging situations (35)	73 (19.6)	84 (22.6)	65 (17.5)	150 (40.3)
Thinking about the donor gives me a positive feeling in various life situations (33)	95 (25.4)	99 (26.5)	59 (15.8)	121 (32.4)
When I think of the donor, I can show my gratitude (30)	206 (54.6)	97 (25.7)	33 (8.8)	41 (10.9)
When I think of the donor, I have the feeling that I am not alone (25)	39 (10.2)	55 (14.4)	80 (20.9)	208 (54.5)
Negative emotions				
The feelings toward the donor depress me (36)	10 (2.7)	32 (8.6)	74 (19.9)	255 (68.7)
I wish I could switch off the thoughts about the donor (38)	11 (3.0)	18 (4.9)	47 (12.7)	293 (79.4)
Sometimes I have the feeling I’m ruminating over the donor (33)	9 (2.4)	40 (10.7)	68 (18.2)	257 (68.7)

#### 3.5.4 Exploratory associations between donor and donation images content factors and perceived consequences

In a first exploratory regression model, we examined whether the three content factors (Social, Sex/Age, Identity) showed any statistical associations with perceived changes in personality and behavior (index comprising six items). The factor Social showed a small but statistically significant association, while Sex/Age and Identity were not associated with this outcome (see Table 8). The model explained only a small proportion of variance (R^2^ = 0.095), indicating limited explanatory value despite statistical significance at the model level (*p* < 0.001).

**Table 8:** Exploratory regression models for associations between DDI content factors and perceived consequences.

Predictor for consequences of DDI	Regression coefficient *B*	Standard error (*SE*)	Significance (*p*)	95% Confidence interval for *B*
Change in personality and behavior				
Constant	0.89	0.07	< 0.001	[0.74, 1.03]
Factor social	0.93	0.17	0.001	[0.60, 1.26]
Factor sex, age	0.05	0.10	0.631	[−0.15, 0.25]
Factor identity	−0.14	0.28	0.624	[−0.69, 0.41]
Influence on organ integration				
Constant	0.53	0.04	< 0.001	[0.45, 0.61]
Factor social	0.02	0.09	0.859	[−0.17, 0.20]
Factor sex, age	0.02	0.06	0.761	[−0.10, 0.13]
Factor identity	0.24	0.15	0.115	[−0.06, 0.53]
Generating positive emotions				
Constant	1.05	0.08	< 0.001	[0.90, 1.20]
Factor social	0.86	0.17	< 0.001	[0.52, 1.19]
Factor sex, age	0.29	0.11	0.006	[0.08, 0.50]
Factor identity	−0.07	0.28	0.797	[−0.63, 0.49]
Generating negative emotions				
Constant	0.22	0.06	< 0.001	[0.12, 0.33]
Factor social	0.51	0.12	< 0.001	[0.28, 0.76]
Factor sex, age	0.12	0.08	0.112	[− 0.03, 0.27]
Factor identity	−0.18	0.20	0.369	[−0.57, 0.21]

DDI = Donor and Donation Images. All models are exploratory and explain only small proportions of variance; results indicate statistical associations rather than predictive effects. Sample sizes varied across models due to missing data: change in personality and behavior, *N* = 376; organ integration, *N* = 385; positive emotions, *N* = 373; negative emotions, *N* = 360.

In the second exploratory model, we examined potential associations between the factors and perceived organ acceptance (six-item index). None of the factors were significantly associated with this outcome (see Table 8). The overall model was not statistically significant (*p* = 0.341) and accounted for only minimal variance (R^2^ = 0.009).

In the third model, we explored associations between the factors and perceived positive emotional consequences (five-item index). The model reached significance (*p* < 0.001), and the factors Social and Sex/Age were associated with perceived positive emotional responses, whereas Identity was not (see Table 8). Still, explained variance remained modest (R^2^ = 0.119).

In the fourth model, we assessed associations with negative emotional consequences (three-item index). The factor Social showed a statistically significant association, while the remaining factors did not. The model explained a small proportion of variance (R^2^ = 0.067).

The Spearman correlation between the factors and the indices are presented in Supplementary Table 3. Overall, all four regression analyses should be interpreted as exploratory. Across outcomes, the models explained only small proportions of variance, indicating that the identified associations are weak and do not reflect predictive or causal relationships.

## 4 Discussion

This study represents the first systematic attempt to quantify the content of DDI across 12 themes (death, age, sex, family situation, life history, personality, lifestyle, origin, profession, education, sexual orientation, religion). We examined perceived emotional consequences, perceived personality and behavioral changes, and perceived organ acceptance, while also exploring gender-associated differences. Given the limited existing knowledge on DDI, all analyses were conducted exploratively and should be interpreted accordingly. Therefore, descriptive findings, exploratory associations, and potential clinical considerations are discussed separately below.

The study was based on a large cohort of 402 individuals who had undergone HTX. Recipients most frequently thought about the donor’s cause of death (66%), age (65%), and sex (60%). Women reported thinking more often about the donor’s sex, age, and personality traits, whereas no significant gender differences emerged for the remaining themes. In line with our analytical focus on gender, previous analyses of the same cohort demonstrated that other sociodemographic variables (such as partnership status) were not significantly associated with DDI (Laskowski et al., 2025).

Although we did not identify HTX-specific evidence addressing gender differences in cognitions related to the donor, findings from adjacent areas of transplant psychology suggest that women may engage more strongly in relational and interpretative meaning-making. For example, in a qualitative study of adult kidney transplant recipients (both living and deceased donation), women described greater emotional attunement to the donor, heightened attention to relational aspects, and more elaborated interpretations of donor characteristics compared to men (Bourkas and Achille, 2023). While such findings cannot be directly transferred to HTX, they may offer a tentative framework for understanding why women in our sample reported more frequent thoughts about selected donor attributes.

Most participants assumed that the donor had died in an accident (78%), although epidemiological data from Germany indicate that traumatic brain injury accounts for only about 15% of donor deaths, with non-traumatic causes such as intracranial hemorrhage (49%) and ischemic stroke (24%) being far more common (Deutsche Stiftung Organtransplantation, 2025). Beyond brain-related causes, national data also show that cardiocirculatory conditions constitute another relevant group of donor causes of death. The leading causes among organ donors include intracranial hemorrhage, ischemic stroke, hypoxic brain injury, and cardiocirculatory collapse (Deutsche Stiftung Organtransplantation, 2025).

Likewise, most recipients (60%) believed their donor to have been younger than themselves. These assumptions align with prior findings but contrast with actual statistics (Frierson and Lippmann, 1987; Sadala and Stolf, 2008; Conway et al., 2013). They may reflect common stereotypes about organ donation or a desire to imagine receiving a “healthy and strong” heart. Thus, recipients may construct detailed representations of an otherwise largely unknown donor despite the absence of identifying information. At the same time, non-identifying donor characteristics may have been known to some recipients. As we did not assess whether participants had received such information, this may have influenced their reported assumptions.

Regarding donor sex, 59% of participants assumed a same-sex donor, while 40% imagined an opposite-sex donor. These assumptions mirror prior qualitative reports (Laskowski et al., 2023) but differ from actual sex-mismatch rates in HTX: in the United States between 2010 and 2018, 16.10% of HTX involved female-to-male and 9.7% male-to-female donor–recipient combinations (Verona et al., 2024). Previous work suggests that imagining a same-sex donor may feel more natural to some recipients, whereas others express either concerns (particularly men) or positive associations (particularly women) related to gendered characteristics (Sanner, 2003). While these interpretations remain speculative, they underline the complexity and individuality with which recipients construct donor-related imagery.

Beyond the descriptive distribution of DDI themes, we explored whether these content dimensions showed any statistical associations with perceived consequences. The 12 content themes clustered into three factors: Social, Sex/Age, and Identity, which were then explored in relation to perceived consequences. The Social factor showed small but statistically significant associations with perceived personality and behavioral changes and with both positive and negative emotional responses. The Sex/Age factor was weakly associated with positive emotions, whereas the Identity factor showed no relevant associations. Across all analyses, however, the explained variance was low, indicating that these associations represent weak relationships and should be interpreted as exploratory.

None of the factors showed meaningful associations with perceived organ acceptance. This pattern is consistent with the descriptive findings: most recipients (81%) reported no difficulty accepting the transplanted heart, and the majority (84%) did not believe that DDI affect this acceptance. From a hypothesis-generating perspective, a smaller subset (25%) reported that DDI facilitated organ acceptance, which (although not the majority) may nonetheless be clinically relevant. For patients who experience uncertainty or distress in the integration process, acknowledging and addressing DDI in clinical conversations could potentially support emotional adjustment and, indirectly, adaptation to the graft. Given that psychological distress and impaired acceptance have been linked to poorer post-transplant adherence and, in some cases, graft complications (Goetzmann et al., 2009; Hennemann et al., 2021), even minor effects may warrant clinical attention.

Prior work has described gratitude as a central emotional experience among HTX recipients (Laskowski et al., 2025), which may offer an important entry point for psychosocial support. Integrating discussions around DDI into routine follow-up care may therefore help address individual concerns, strengthen coping processes, and ultimately contribute to improving long-term well-being in this highly vulnerable patient group.

## 5 Limitations

Several limitations of this study must be acknowledged. First, the cross-sectional design precludes any causal inference. All associations identified should therefore be interpreted as exploratory and descriptive rather than predictive or explanatory.

Second, the study relied on a self-developed questionnaire, as no standardized instrument for assessing DDI currently exists. Although this allowed us to systematically map a wide range of themes, the measure has not undergone formal psychometric validation. Moreover, the questionnaire covered 12 predefined categories drawn from earlier qualitative studies; therefore, additional relevant themes may not have been captured. In addition, the dichotomous response format constrained variance and likely limited factor stability, which is reflected in the tentative nature of the extracted factor structure and the reduced internal consistency of the Identity factor. Future work should refine, update, and validate DDI assessment tools to reflect contemporary transplant experiences and psychosocial contexts.

Third, the time since HTX varied substantially within the sample, with some respondents recalling events and impressions from many years ago. This variability introduces potential recall bias. Moreover, we did not assess whether recipients had received non-identifying information about their donors, such as donor sex or age. Consequently, we cannot determine the extent to which reported assumptions reflect imagined versus previously known donor characteristics.

Fourth, recruitment from a single major transplant center may limit generalizability, and self-selection processes may have influenced participation. Individuals with substantial medical or psychological difficulties may have been unable or unwilling to participate, potentially leading to underrepresentation of those for whom DDI are most distressing.

Fifth, the explained variance of all regression models was low. This indicates that the extracted factors account for only a small proportion of variability in perceived consequences and that substantial unmeasured factors may likely play a role. Accordingly, the observed associations should not be interpreted as clinically meaningful predictors. Moreover, physiological factors such as side effects of immunosuppressive medication (e.g., tacrolimus-related irritability) (Verona et al., 2024), corticosteroid-related agitation (Nieman, 2015; National Institute of Diabetes and Digestive and Kidney Diseases, 2021) may contribute to perceived personality or emotional changes and could not be disentangled from psychological processes in the present study.

Sixth, the study is subject to survivorship bias: only recipients who survived HTX were able to participate. Consequently, perspectives of individuals who died shortly after transplantation are not represented. Prior research in kidney transplantation suggests that organ acceptance is strongly shaped by social support, graft function, and time since transplantation (Hennemann et al., 2021). Given that 19% of our sample had undergone HTX more than 20 years ago, the high levels of reported organ acceptance in this cohort may partly reflect long-term graft stability and sustained adaptation rather than the absence of DDI effects.

## 6 Conclusion

Overall, this study provides an initial, exploratory overview of DDI content in a large cohort of HTX recipients. Due to the cross-sectional design, the use of a non-validated instrument, potential recall and survivorship biases, and the limited explanatory power of the statistical models, the findings should be considered preliminary. Longitudinal research, standardized and validated assessment tools, and multimethod designs will be essential to clarify how DDI form, how they change over time, and how they relate to psychosocial well-being and clinical outcomes.

## Data Availability

The raw data supporting the conclusions of this article will be made available by the authors upon reasonable request.
