# Supplementary material for: My heart will go on – quantitative content analysis of donor and donation images in patients after heart transplantation
Source: Front Psychol. 2026 Sep 8;17:1813966. doi: 10.3389/fpsyg.2026.1813966 (PMC13595842; doi:10.3389/fpsyg.2026.1813966)
Supplement: Supplementary file 1 [file Supplementary_file_1.docx]

Supplementary Material

# 1 Supplementary Tables

Supplementary Table 1: Tetrachoric correlation between the content categories of Donor and Donation Images

|  |  | **DDI content categories** | | | | | | | | | | |
| --- | --- | --- | --- | --- | --- | --- | --- | --- | --- | --- | --- | --- |
|  |  | Age | Sexual Orientation | Origin | Religion | Personality | Life History | Lifestyle | Profession | Education | Family Situation | Death of the Donor |
| **DDI content categories** | Sex | 0.65 | 0.18 | 0.26 | 0.08 | 0.27 | 0.28 | 0.26 | 0.16 | 0.21 | 0.32 | 0.16 |
|  | Age | - | 0.16 | 0.21 | 0.07 | 0.23 | 0.25 | 0.23 | 0.13 | 0.18 | 0.28 | 0.15 |
|  | Sexual Orientation | - | - | 0.19 | 0.46 | 0.14 | 0.13 | 0.27 | 0.20 | 0.29 | 0.14 | 0.04 |
|  | Origin | - | - | - | 0.20 | 0.21 | 0.20 | 0.23 | 0.20 | 0.21 | 0.25 | 0.03 |
|  | Religion | - | - | - | - | 0.21 | 0.15 | 0.16 | 0.33 | 0.22 | 0.15 | 0.04 |
|  | Personality | - | - | - | - | - | 0.40 | 0.34 | 0.40 | 0.47 | 0.37 | 0.03 |
|  | Life History | - | - | - | - | - | - | 0.43 | 0.41 | 0.39 | 0.54 | 0.17 |
|  | Lifestyle | - | - | - | - | - | - | - | 0.35 | 0.46 | 0.46 | 0.10 |
|  | Profession | - | - | - | - | - | - | - | - | 0.63 | 0.33 | 0.08 |
|  | Education | - | - | - | - | - | - | - | - | - | 0.28 | 0.05 |
|  | Family Situation | - | - | - | - | - | - | - | - | - | - | 0.14 |

*Note. DDI = Donor and Donation Images; all correlations are significant at* p*<.001.*

Supplementary Table 2: Measure of Sampling Adequacy for content categories of Donor and Donation Images

| **DDI content categories** | **Measure of Sampling Adequacy (MSA)** |
| --- | --- |
| Sex | 0.71 |
| Age | 0.70 |
| Sexual Orientation | 0.68 |
| Origin | 0.91 |
| Religion | 0.66 |
| Personality | 0.88 |
| Life History | 0.84 |
| Lifestyle | 0.89 |
| Profession | 0.78 |
| Education | 0.77 |
| Family Situation | 0.85 |
| Death | 0.81 |

*Notes. DDI = Donor and Donation Images.*

Supplementary Table 3: Spearman’s correlation between factors and indices

| **Factors** |  | **Factors (Content of DDI)** | | | **Indices (Consequences of DDI)** | | | |
| --- | --- | --- | --- | --- | --- | --- | --- | --- |
|  |  | Social | Body | Identity | Change in personality/behavior | Organ Integration | Positive Emotions | Negative Emotions |
|  | Social | - | 0.42** | 0.24** | 0.31** | 0.01 | 0.33** | 0.28** |
|  | Body | - | - | 0.17** | 0.15** | 0.04 | 0.24** | 0.16** |
|  | Identity | - | - | - | 0.06 | 0.08 | 0.09 | 0.03 |
| **Indices** | Change in personality/behavior | - | - | - | - | 0.15** | 0.32** | 0.20** |
|  | Organ Integration | - | - | - | - | - | 0.07 | 0.26** |
|  | Positive Emotions | - | - | - | - | - | - | 0.29** |

*Note. Two-sided; **correlations significant at p<.001.*
